# Supplementary material for: Radiation-Induced EMT of Adipose-Derived Stem Cells in 3D Organotypic Culture via Notch Signaling Pathway
Source: Biology (Basel). 2025 Sep 22;14(9):1306. doi: 10.3390/biology14091306 (PMC12467909; doi:10.3390/biology14091306)
Supplement: Supplementary file 1 [file biology-14-01306-s001.zip › biology-3846537-supplementary/biology-3846537-supplementary-table S1.pdf]

Supplementary Table S1. Primer sequences for Reverse Transcription Quantitative PCR

Primers		Sequences
mouse GAPDH	Forward	5'-CACTCACGGCAAATTCAACG-3'
	Reverse	5'-GACTCCACGACATACTCAGC-3'
mouse α-SMA	Forward	5'-GGACGTACAACTGGTATTGTGC-3'
	Reverse	5'-GGATCTTCATGAGGTAGTCGG-3'
mouse Fibronectin	Forward	5'-GAGACTTCTCTCCTCAATGGTG-3'
	Reverse	5'-CCTATTGATCCCAGACCAAACC-3'
mouse N-cadherin	Forward	5'-CATCATCACTGTGGCAGCTG-3'
	Reverse	5'-GCTGTGGCTGTGTTTGAAAG-3'
mouse Vimentin	Forward	5'-TGCGCCAGCAGTATGAAA-3'
	Reverse	5'-CCGGTACTCGTTTGACTCCTG-3'
mouse ZO-1	Forward	5'-GCAGAGACAATAGCATTCTCCC-3'
	Reverse	5'-GGTTTTAGGGTCACAGTGTGG-3'
mouse CK-18	Forward	5'-CAGCGTCTATGCAGGTGC-3'
	Reverse	5'-GGTCTTGCATGGTCTCCTTC-3'
mouse EpCAM	Forward	5'-CTGAGAGTGAACGGAGAGC-3'
	Reverse	5'-GCGATGACTGCTAATGACACC-3'
mouse E-cadherin	Forward	5'-GCTCTCATCATCGCCACAG-3'
	Reverse	5'-GATATGAGGCTGTGGGTTCC-3'
mouse Slug	Forward	5'-CCAAGAAGCCCAACTACAGC-3'
	Reverse	5'-GTAATAGGGCTGTATGCTCCC-3'
mouse Twist1	Forward	5'-GTGGACAGAGATTCCCAGAG-3'
	Reverse	5'-CTTCGTCAAAAAGTGGGGTGG-3'
mouse Twist2	Forward	5'-GCAAGATCCAGACGCTCAAG-3'
	Reverse	5'-AGAAGGCGTAGCTGAGAC-3'

Primers		Sequences
mouse Snail	Forward	5'-CCACTGCAACCGTGCTTTT-3'
	Reverse	5'-AAGGACATGCGGGAGAAG-3'
mouse Notch1	Forward	5'-GTGCTCTGATGGACGACAAT-3'
	Reverse	5'-GTCTGATCACTCAGGTCAGG-3'
mouse Notch2	Forward	5'-GAGAGGACTGCCAATACTC-3'
	Reverse	5'-AGGTGCACTCGTAGGTGT-3'
mouse Notch3	Forward	5'-GATTCTCCTGTCGTTGTCTC-3'
	Reverse	5'-CAGCTTTGACCCTGGTAG-3'
mouse Notch4	Forward	5'-CTGTGAGGTGGAGGTCAATG-3'
	Reverse	5'-CCATGTCTTTCTCACATCGG-3'
mouse Jagged1	Forward	5'-CTTGGGTCTGTTGCTTGGTG-3'
	Reverse	5'-TGGCTCCGTGTTTCTCGATG-3'
mouse Jagged2	Forward	5'-CACCGGGATTGTAGCAAGGT-3'
	Reverse	5'-CCTTCTCCTGACATTGCTGC-3'
mouse DLL1	Forward	5'-GTTCAGATAACCCTGACGGAG-3'
	Reverse	5'-CACACTTGGCACCGTTAGAAC-3'
mouse DLL3	Forward	5'-GGATCCACAGCGCTTTCTTC-3'
	Reverse	5'-CAGGTTGTTGAGTGCATCCG-3'
mouse DLL4	Forward	5'-GAATGTATCCCCCACAATGG-3'
	Reverse	5'-CACTGTTGGAACACGTTGATCC-3'
mouse STAT3	Forward	5'-CCTGTGGTATAACATGCTGACC-3'
	Reverse	5'-CTCTACACAGACTGGATCTGGG-3'
mouse NF-κB	Forward	5'-GAATGGCTCGTCTGTAGTG-3'
	Reverse	5'-TGGTATCTGTGCTCCTCTC-3'
mouse Fra-1	Forward	5'-GCTGCAGAAGCAGAAGGAAC-3'
	Reverse	5'-GTACGGGTCCTGGAGAAAG-3'
