# Supplementary material for: Radiation-Induced EMT of Adipose-Derived Stem Cells in 3D Organotypic Culture via Notch Signaling Pathway
Source: Biology (Basel). 2025 Sep 22;14(9):1306. doi: 10.3390/biology14091306 (PMC12467909; doi:10.3390/biology14091306)
Supplement: Supplementary file 1 [file biology-14-01306-s001.zip › biology-3846537-supplementary/biology-3846537-supplementary-WB Figure S1.pdf]

Figure S1. Western blot analysis of E-cadherin (120 kDa) and vimentin (57 kDa) expression in 3D organoids and 2D ASCs, respectively. GAPDH (37 kDa) served as a loading control. Original blot images corresponding to Figure 2C are provided.

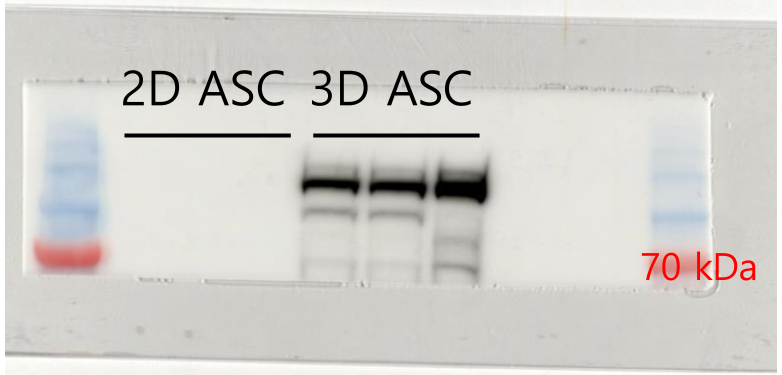

E-cadherin

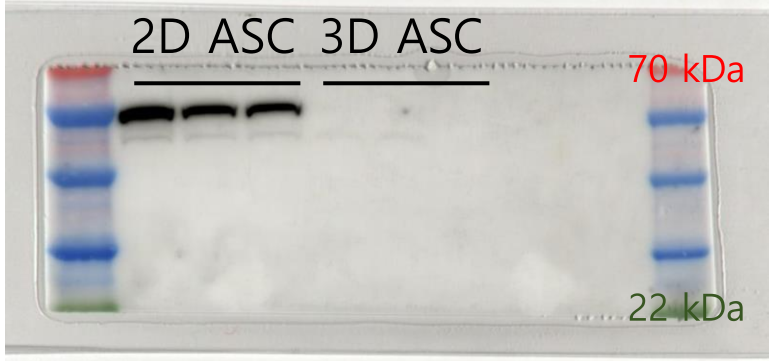

Vimentin

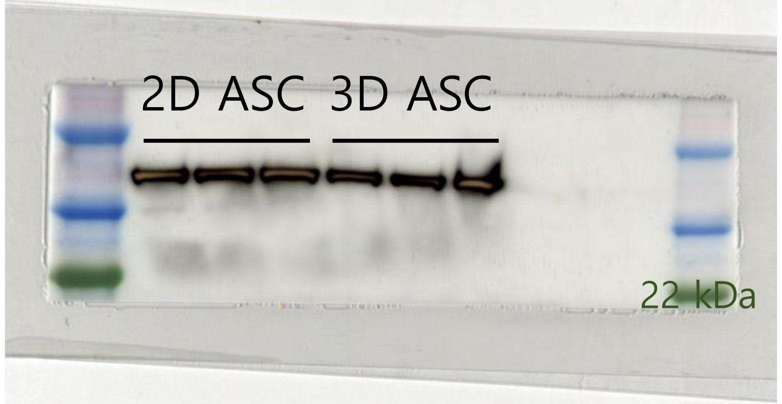

GAPDH
