# Supplementary material for: Radiation-Induced EMT of Adipose-Derived Stem Cells in 3D Organotypic Culture via Notch Signaling Pathway
Source: Biology (Basel). 2025 Sep 22;14(9):1306. doi: 10.3390/biology14091306 (PMC12467909; doi:10.3390/biology14091306)

Figure S2. Western blot analysis of E-cadherin and fibronectin protein levels in 2D ASCs, 3D organoids, irradiated 3D organoids, and GSI-pretreated irradiated organoids. GAPDH served as a loading control. Original blot images corresponding to Figure 6B are provided, and part of the data also covers the experiments presented in Figure 4C.

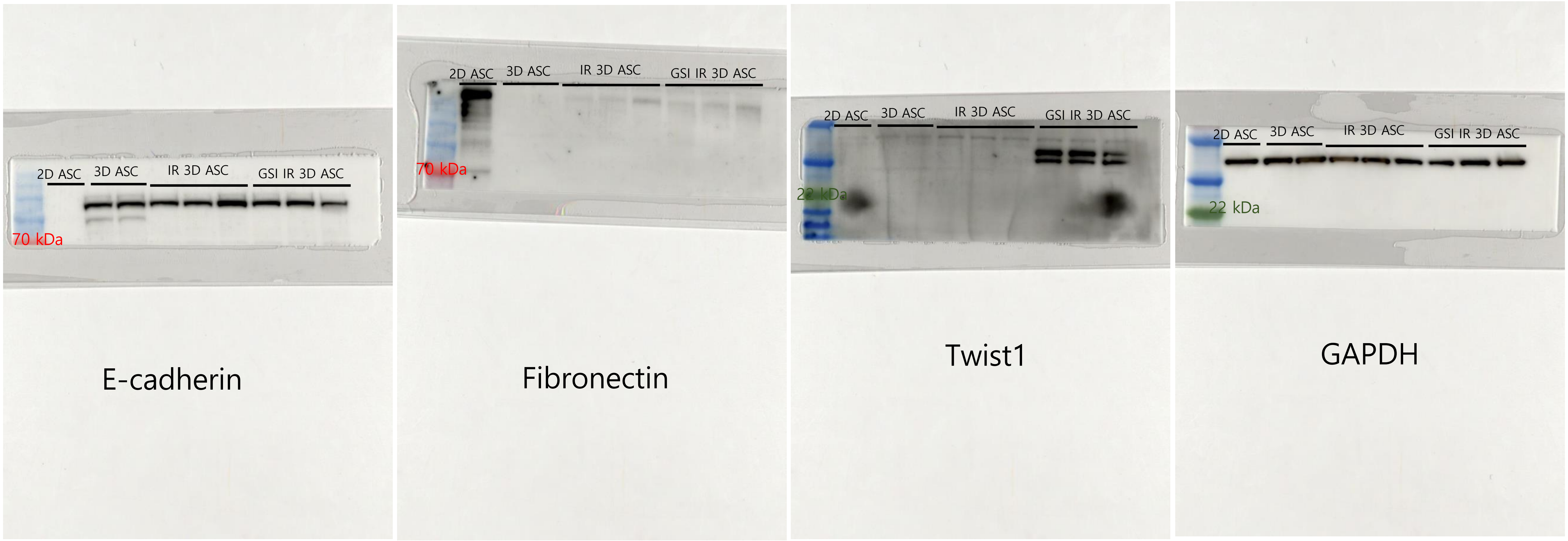

Supplement: Supplementary file 1 [file biology-14-01306-s001.zip › biology-3846537-supplementary/biology-3846537-supplementary-WB Figure S2.pdf]
